# Sphingolipid Signature of Human Feto-Placental Vasculature in Preeclampsia

**DOI:** 10.3390/ijms21031019

**Published:** 2020-02-04

**Authors:** Ilaria Del Gaudio, Linda Sasset, Annarita Di Lorenzo, Christian Wadsack

**Affiliations:** 1 Department of Obstetrics and Gynecology, Medical University of Graz, 8036 Graz, Austria; ilaria.del-gaudio@medunigraz.at; 2 Department of Pathology and Laboratory Medicine, Cardiovascular Research Institute, Feil Family Brain and Mind Research Institute, Weill Cornell Medical College, Cornell University, New York, NY 10065, USA

**Keywords:** sphingolipid metabolism, bioactive lipids, human placenta, placental vasculature, preeclampsia, feto-placental endothelium

## Abstract

Bioactive sphingolipids are emerging as key regulators of vascular function and homeostasis. While most of the clinical studies have been devoted to profile circulating sphingolipids in maternal plasma, little is known about the role of the sphingolipid at the feto-placental vasculature, which is in direct contact with the offspring circulation. Our study aims to compare the sphingolipid profile of normal with preeclamptic (PE) placental chorionic arteries and isolated endothelial cells, with the goal of unveiling potential underlying pathomechanisms in the vasculature. Dihydrosphingosine and sphingomyelin (SM) concentrations (C16:0-, C18:0-, and C24:0- sphingomyelin) were significantly increased in chorionic arteries of preeclamptic placentas, whereas total ceramide, although showing a downward trend, were not statistically different. Moreover, RNA and immunofluorescence analysis showed impaired sphingosine-1-phosphate (S1P) synthesis and signaling in PE vessels. Our data reveal that the exposure to a deranged maternal intrauterine environment during PE alters the sphingolipid signature and gene expression on the fetal side of the placental vasculature. This pathological remodeling consists in increased serine palmitoyltransferase (SPT) activity and SM accrual in PE chorionic arteries, with concomitance impairment endothelial S1P signaling in the endothelium of these vessels. The increase of endothelial S1P phosphatase, lyase and S1PR2, and blunted S1PR1 expression support the onset of the pathological phenotype in chorionic arteries.

## 1. Introduction

Preeclampsia (PE) is characterized by profound morphological and functional modifications in the arterial vessels of the uterus and the placenta. Together, poor vascular adaptions in the mother and an inflammatory intrauterine environment during pregnancy, affect the functionality of the feto-placental endothelium. This does not only lead to pregnancy-related maternal and fetal morbidities but also to adverse outcomes for the offspring later in life, e.g., increased risk to develop hypertension (3-fold) and cardiovascular disease (2-fold) later in life [1,2,3]. PE has an incidence of 3–5% of pregnancies in the United States and up to 10% of pregnancies worldwide [4], representing the leading cause of maternal and fetal morbidity and mortality. Clinically, PE is defined as the de novo onset of hypertension (systolic blood pressure > 140 mmHg or diastolic blood pressure > 90 mmHg) and proteinuria (>300 mg/24 h) after 20 to 24 weeks of gestation [5]. The symptoms of PE typically remain until delivery and are reversed after delivery, when blood pressure levels return to pre-pregnancy levels. Although the specific mechanisms leading to the development of PE have to be elucidated yet, the indicators of the endothelial dysfunction in PE often synergize, exacerbating the condition. For instance, an association between impaired lipid metabolism and loss of endothelium functionality has been proposed in women who develop PE [6].

Sphingolipids, resulting from the de novo biosynthesis in the endoplasmatic reticulum (ER) or from the catabolism of complex sphingolipids and recycling pathway [7], have emerged as a class of bioactive lipids that play an important role in vascular homeostasis. Among the sphingolipids, ceramide (Cer) and sphingosine-1-phosphate (S1P) can differentially regulate endothelial functions. Once synthesized, mainly in red blood cells and vascular endothelium [8], S1P is rapidly exported out of the cells, where it can activate cell-surface receptors (S1PRs) in an autocrine fashion or can bind plasma chaperones. Approximately 65% of circulating S1P is bound to high density lipoprotein (HDL), while the remaining 35% associates to albumin [9]. S1P-mediated S1PR1 activation enhances cellular proliferation, survival, and nitric oxide (NO) production, resulting in blood vessel relaxation and atheroprotection [10,11]. Interestingly, plasma levels of S1P are significantly diminished in patients affected by myocardial infarction and coronary artery disease [12,13].

Multiple studies reported a correlation between Cer imbalance and cardiovascular diseases as well as metabolic disorders [14,15].

Sphingomyelin (SM), complex sphingolipid found in plasma and cellular membranes, plays a pivotal role in membrane stability and cell cholesterol homeostasis [16]. Several studies demonstrated an association between altered SM levels and cardiovascular diseases [17].

Whereas a substantial research effort investigated the impact of altered sphingolipids’ metabolism and their role in the pathogenesis of cardiovascular diseases, their importance in pregnancy and the function of the placenta, a highly vascularized organ, remains poorly understood. It has been shown that there is a derangement in sphingolipid metabolism and levels in the human term umbilical cord artery (UCA) [18] and plasma of patients diagnosed with PE [19,20].

The aim of this study is to investigate whether PE affects the key players of the sphingolipid metabolism to impact their signature in the feto-placental vasculature, which plays a critical role in regulating angiogenesis, vasomotor tone, and placental perfusion, pivotal to fetal development.

## 2. Results

### 2.1. Sphingolipid Profile of Placental Chorionic Arteries from PE and Normotensive Subjects

**Figure 1:**
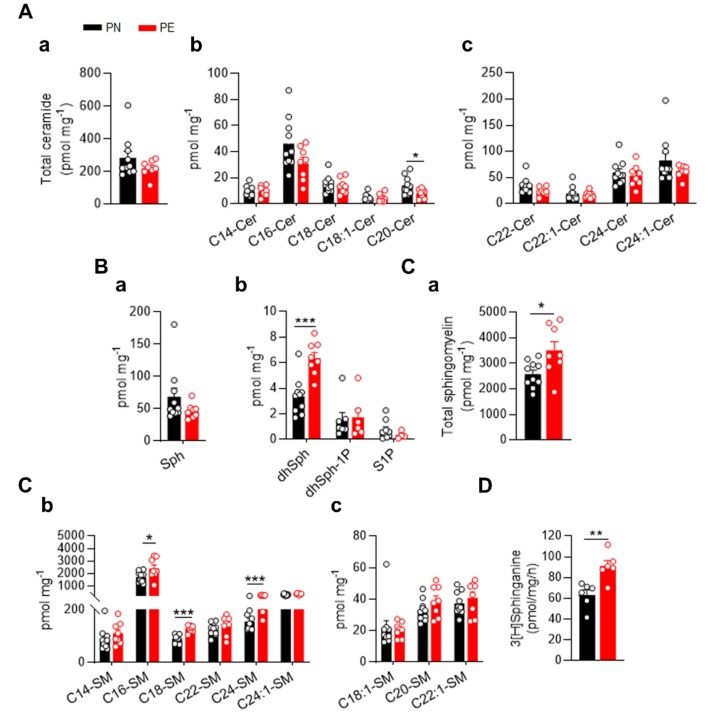
Sphingolipids content and serine palmitoyltransferase (SPT) activity of normotensive (PN) and preeclamptic (PE) chorionic arteries. LC-MS quantification of ceramide (**A**), sphingosine intermediates (**B**), and sphingomyelin (**C**) of isolated placental chorionic arteries from normotensive (*n* = 10) and preeclamptic women (*n* = 8). Total (**A**, **a**) and individual (**A**, **b**; **A**, **c**) ceramide species. (**B**) Dihydrosphingosine (dhSph), sphingosine (Sph), dihydrosphingosine -1-phosphate (dhSph-1P), and S1P. Total (**C**, **a**) and individual (**C**, **b**; **C**, **c**) sphingomyelin species. (**D**) Chorionic arteries lysates were assessed for SPT activity. [3H]-serine and palmitoyl-CoA were used as substrates by SPT to generate 3-ketosphinganine, subsequently reduced to sphinganine, followed by TLC separation. Sphinganine was used as the marker (*n* = 6 per group). Data are expressed as mean ± SEM * *P* < 0.05; ** *P* < 0.01 *** *P* < 0.001 compared to PN. Statistical significance was determined by unpaired *t*-test.

It has been reported that sphingolipid signaling plays an important role in the vascular function and blood pressure homeostasis [21]. Considering that hypertension and the endothelial dysfunction are major hallmarks of PE, we reasoned that sphingolipid metabolism might play a role in the PE-induced placental vascular dysfunction. Thus, we determined the sphingolipid levels in chorionic placental arteries from normotensive (PN) and PE pregnancies. Interestingly, LC-MS/MS analysis showed a decreasing trend in total Cer levels in PE arteries compared to heathy subjects, although this was not statistically significant (Figure 1A(a)). The quantification of single ceramide species showed that, whereas an overall trend towards reduced levels could be observed in different species, only C20:0-cer was significantly decreased in PE compared to PN chorionic arteries (Figure 1A(b),(c)).

Interestingly, sphingosine and S1P, downstream products of ceramide, also presented a decreasing trend in PE arteries versus control patients, although this was not significant (Figure 1B(a),(b)). Notably, the level of dihydrosphingosine (dhSph), a downstream product of serine palmitoyltransferase (SPT), the first and rate limiting enzyme of the de novo biosynthesis, was significantly augmented in chorionic arteries of preeclamptic women (Figure 1B(b)), suggesting an upregulation of this pathway. The dhSph levels have been reported to be associated with cardiovascular disease [22]. Accordingly, PE was accompanied by an accrual of total SM levels (Figure 1C(a)). The analysis of the single SM species revealed a significantly higher content of C16:0-, C18:0-, and C24:0-cer in PE arteries compared to PN (Figure 1C(b)). These data collectively suggest that during PE, the sphingolipid metabolism of the feto-placental vasculature is shifted towards SM, by altered production/catabolism.

The de novo sphingolipid synthesis takes place at the ER through the action of SPT, encoded by the genes SPTLC1 and SPTLC2) [23]. To corroborate an upregulation of SL biosynthesis, the SPT enzymatic assay was performed as previously reported [21]. As shown in Figure 1D, SPT activity was significantly increased in PE versus PN chorionic arteries, in agreement with the sphingolipid profile.

### 2.2. PE Impairs S1P Signaling at the Feto-Placental Vasculature

The endothelium is a major source of S1P. This bioactive lipid is a potent regulator of vascular integrity, due to its ability to enhance the endothelial barrier function, induce the production of NO and exert anti-inflammatory and anti-atherogenic effects [9,10,24,25]. Disruption of S1P metabolism has been implicated in many cardiovascular diseases, in which the endothelial dysfunction represents a common denominator [26].

Within the vascular bed, endothelial cells can produce and release (via spinster 2 transporter, SPNS2) S1P [8]. Its intracellular levels are tightly regulated by sphingosine kinases (*SPHK1* and *SPHK2*) and degrading enzymes such as S1P phosphatase (*SGPP1*) and sphingosine-1-phosphate lyase (*SGPL1*) [27]. Once S1P is secreted out of the cell it may signal via G protein-coupled receptors (GPCRs) via S1PR1, S1PR2, and S1PR3, which are all expressed in the vasculature [28]. We have previously reported that Nogo-B (encoded by the gene *RTN4*) is a negative regulator of SPT activity, highly expressed in the endothelium of blood vessels [21]. Mice lacking endothelial Nogo-B are protected by hypertension and heart failure [29] via upregulation of S1P-S1PR1-NO signaling [21], suggesting the Nogo-B-mediated inhibition of SPT plays a pathological role in the onset of cardiovascular diseases.

**Figure 2:**
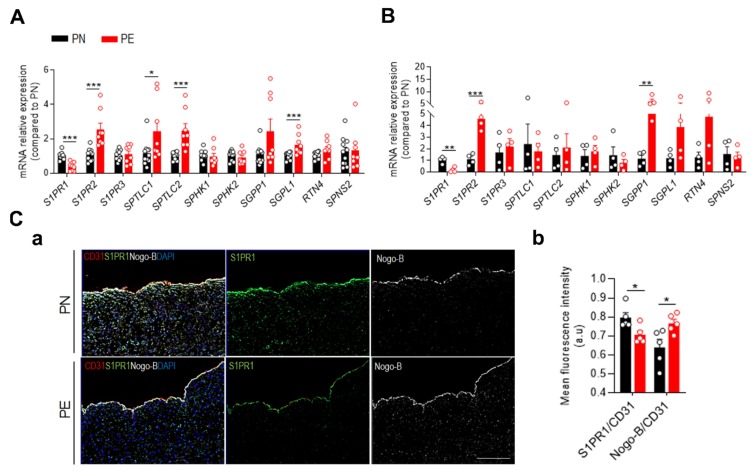
PE alters S1P signaling. RT-PCR of PN (*n* = 10) and PE (*n* = 8) homogenates of chorionic arteries (**A**) and isolated fPAEs (PN *n* = 4; PE *n* = 4) (**B**). Representative immunofluorescence staining of CD31, S1PR1 and Nogo-B in placental chorionic arteries of PN (*n* = 5) and PE (*n* = 5) subjects. Scale bar: 100 µm (**C**, **a**). Scatter plot of fluorescence intensity quantified by using ImageJ. Mean fluorescence intensity was calculated as the ratio of S1PR1/CD31 and Nogo-B/CD31. CD31 was used as the reference marker for the endothelium (**C**, **b**). Data are expressed as mean ± SEM * *P* < 0.05; ** *P* < 0.01; *** *P* < 0.001 compared to PN. Statistical significance was determined by unpaired *t*-test.

To assess the effect of PE on S1P signaling in the human placenta, we evaluated the expression of key genes involved in the sphingolipid pathway in placental chorionic arteries (Figure 2A) and fetal placental arterial endothelial cells (fPAECs) (Figure 2B). RNA analysis revealed that PE caused an increased expression of SPTLC1 and SPTLC2, suggesting an increased sphingolipid biosynthesis in the arteries of PE subjects (Figure 2A). The expression of the sphingosine kinases was not different suggesting that the formation of S1P is not impaired in the whole vessel. However, the amount of Sph in PE arteries is reduced, although not statistically different, indicating that the limited substrate of SPHK1/2 might contribute to decreased S1P production. PE chorionic arteries showed also a significant increase in *SGPL1* expression, a degrading enzyme of S1P [30]. These data suggest that, despite the increased sphingolipid biosynthesis and SM, S1P production might be impaired.

Notably, the expression of *S1PR1* was significantly downregulated, whereas *S1PR2* levels were increased in isolated chorionic arteries from PE subjects compared to controls. These data corroborate the concept that S1P can induce opposing effects according to the expression levels of the respective receptors involved in the signaling cascade. S1PR1 activation is often associated with vascular protection [31,32,33]. Conversely, induction of S1PR2 has been related to diabetes [34] whereas expression of S1PR3 was unchanged.

Considering that the endothelium is one of the major cellular sources of S1P, and an important regulator of vascular tone and barrier, we next examined the gene expression in fPAECs from PE and PN patients (Figure 2B). Consistent with the findings obtained from the chorionic arteries, fPAEC of PE showed increased expression of SGPP1 and SGPL1, although only the latter was not statistically significant. These findings suggest an impaired endothelial-derived S1P. Interestingly, Nogo-B was upregulated at mRNA levels in fPAEC and at the protein level in the endothelium of PE chorionic arteries versus controls (Figure 2B,C(a),(b)).

Furthermore, mRNA expression of S1PR1 and S1PR2 in the endothelium mirrored the same pattern described in the vessels, with the decrease of the former and increase of the latter (Figure 2B). Accordingly, immunofluorescence staining showed significant diminished levels of S1PR1 in the endothelium of PE chorionic arteries (Figure 2C(a),(b)). These findings suggest that PE impairs S1P production and/or degradation, hence S1PR1 signaling.

## 3. Discussion

Altered sphingolipid metabolism has been associated with the pathogenesis of a repertoire of cardiovascular and metabolic diseases [26,35]. However, the role of sphingolipid metabolism and signaling in pregnancy and pregnancy-related disorders is understudied. Several reports highlighted the importance of sphingolipids in different aspects of the female reproductive system such as uterine decidualization [36], placental trophoblast differentiation [37], and uterine and placental angiogenesis [38]. In the context of PE, most of the studies focused on identifying potential biomarkers in plasma with conflicting results [20,39]. However, current knowledge of the role of sphingolipids in the pathophysiology of placental blood vessels remains elusive.

It is well established that the feto-placental vasculature plays an important role during fetal development. Indeed, fetal growth anomalies can occur despite a normal maternal uteroplacental perfusion, suggesting that a proper function of the feto-placental vasculature unit is mandatory independently of the maternal environment [40]. Our study reveals, for the first time, a pathological sphingolipid remodeling associated with gene profile changes of the placental chorionic plate in PE patients. Similarly, to what Romanowicz et al. found in UCA, we observed that PE was associated with a slight decrease in total ceramide levels [41], with only the reduction of C20:0-Cer levels being statistically significant. On the contrary, the analysis by Melland-Smith et al. revealed a significant increase in the concentration of different ceramide species including C18:0, C20:0, and C24:0 in preeclamptic placentae tissue compared to control [19]. This discrepancy could be explained by the different type of tissues used for the analysis of ceramides. Whereas they used the whole placenta tissues, in our study we focused on the contribution of the feto-placental vasculature, specifically we dissected chorionic arteries. It is also well accepted that sphingolipid metabolism is regulated in a cell type and/or context dependent fashion [7]. Additionally, different pathological mechanisms originated at the maternal side of the placenta might be accountable for the mild decrease in ceramide levels in PE. It is generally accepted that different acyl chains and/or double bonds confer specific biological properties to ceramide species [42]. For instance, augmented levels of C16:0, C18:0, and C20:0 have been associated with anti-proliferative processes and apoptosis [43], whereas very long chain ceramides, such as C24:0, play an anti-apoptotic role [44]. Recently, multiple clinical studies demonstrated a robust correlation between specific plasma ceramide ratios and the occurrence of major cardiovascular events. Peterson et al. reported that higher plasma C24:0/C16:0 ratio negatively correlate with the increased risk of adverse cardiovascular events in patients affected by CAD [45], suggesting that specific changes in the plasma ceramide profile might be indicative of distinct pathological processes. However, measurements of sphingolipids in tissue and plasma samples from preeclamptic donors performed by our group, and others [20,39,46], did not correlate with PE conditions. On the contrary, we found a marked increase in SPT activity, dhSPh and SM, suggesting that a different remodeling of sphingolipid profile occurs during PE compared to CAD. The significant increase in dhSPh measured by LC/MS was strongly supported by the heightened SPT activity of chorionic arteries of PE patients. Increased dhSph levels have been associated with cellular lipotoxicity, in the context of diabetes and neurodegenerative and cardiovascular diseases [47,48,49], and most likely also contributes to the onset of the endothelial dysfunction of the chorionic arteries.

Disruption of SM homeostasis has been also linked to an adverse cardiovascular outcome [17]. Recent studies have demonstrated a positive correlation between serum SM levels and insulin resistance and inflammation [50]. Higher content of SM has been found in UCA as well as in syncytiotrophoblast-derived microvesicles of placentae of preeclamptic women [18,51]. In line with these previous reports, our lipidomic analysis showed a significant upregulation of C16:0-, C18:0-, and C24:0-SM in PE chorionic arteries compared to PN subjects. Notably, the increase in C18:0-SM was also reported in the apical membrane of lipid rafts from placentae as well as in plasma of preeclamptic patients during late gestation [20,52,53]. Considering the increased SM levels in tissue and plasma during preeclampsia [20,52,53], together with our findings of SM increase in PE chorionic arteries, it is tempting to speculate that altered SM content might play a role in the pathogenesis of this syndrome, although further studies need to be performed to validate this conclusion.

Sphingolipids have been implicated in processes regulating the endothelial barrier function and vascular tone [54,55,56]. S1P can enhance the endothelial barrier function [57] and reduce vascular tone by stimulating eNOS-derived NO production [56,58] These effects are mainly mediated by the endothelial S1PR1. Indeed, mice lacking endothelial S1PR1 are hypertensive and present blunted blood flow regulation [31]. Recent studies investigating the role of S1P signaling in pregnancy have demonstrated that the SPHK1/S1P/S1PR1 axis is crucial in early gestation to stimulate placental angiogenesis and the endothelial barrier function during pregnancy [59,60].

Notably, a study conducted by Dobierzewska et al. showed that PE induced a downregulation SPHK1 and S1PR1/3 expression in term placentae [46]. Our data corroborate in part these findings, with an increased expression of SGPL1, SGPP1, and S1PR2 in endothelial cells derived from chorionic arteries, and a concomitant reduction of S1PR1, both at mRNA and protein levels in the endothelium of preeclamptic chorionic arteries compared to controls. These results suggest a decreased endothelial-derived S1P, with a shift of the S1P signaling from S1PR1 to S1PR2-mediated functions. The upregulation of S1PR2 expression occurs in both culture endothelial cells and chorionic artery from PE patients compared to controls. S1PR2, which is described as a pro-inflammatory receptor, promotes the endothelial dysfunction in several pathological conditions [61,62]. Moreover, it mediates the contraction of diverse types of smooth muscle cells. Its activation has been associated with increased pulmonary vascular resistance [63]. Thus, the inverse regulation of S1PR1 and S1PR2 expression in chorionic arteries might explain, at least in part, the vascular dysfunction reported in preeclampsia, including a heightened placental vascular resistance.

Furthermore, the expression of key enzymes involved in the sphingolipid de novo biosynthesis, SPTLC1 and SPTLC2, were significantly increased, as reported in inflammatory and hypertensive [29,64,65]. Accordingly, we found a significant upregulation of SPT activity in chorionic arteries of PE compared to healthy subjects. Interestingly, the increased activity of SPT did not result in the increase of all the sphingolipid subclasses. For instance, total ceramide levels were only slightly reduced, whereas SM significantly accumulated in PE arteries. This is not surprising because multiple enzymes of this metabolic pathway, and their expression levels and post-translational modification, can dictate the sphingolipid landscape in specific cell types, and hence tissues and organs.

We discovered that Nogo-B negatively regulates SPT activity, thereby controlling the de novo sphingolipid biosynthesis [21]. Mice lacking Nogo-B specifically in the endothelium are protected from hypertension and heart failure [21,29], mainly via the upregulation of endothelial-derived S1P S1PR1 signaling. Interestingly, Nogo-B expression was upregulated in the endothelium of preeclamptic chorionic arteries, as well as in the fPAECs. While it is difficult to correlate the SPT activity in the whole chorionic arteries with endothelial Nogo-B, since the expression of Nogo-B is very low in smooth muscle cells compared to the endothelium, our data suggest that Nogo-B upregulation in the endothelium of the feto-placental vasculature might play a role in the pathogenesis PE.

**Figure 3:**
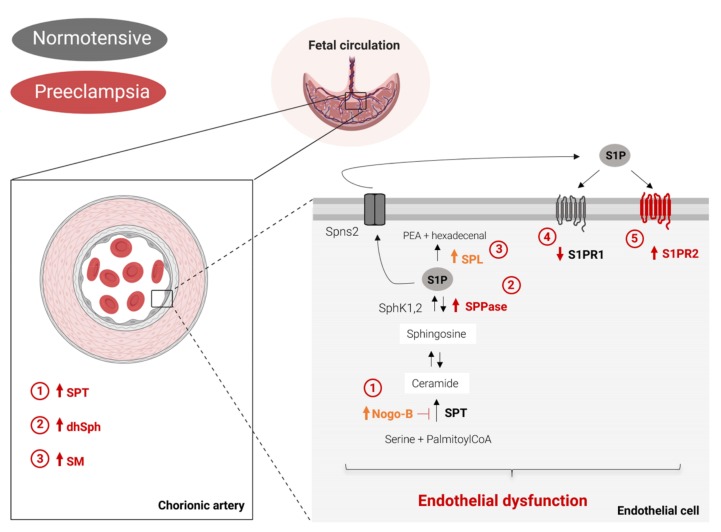
Summary scheme of PE-associated alterations of sphingolipid metabolism and signaling at the feto-placental vasculature. Left, PE is accompanied by increased SPT activity, accumulation of dhSph and increased SM production in chorionic arteries. Right, PE impairs the endothelial function by upregulating the expression of Nogo-B, SPPase, SPL and S1PR2, whereas it reduces the level of S1PR1 (statistically significant changes are depicted in red, whereas not statistically significant alterations are outlined in orange). SPT: serine palmitoyl transferase. KSR: 3-keto-dihydrosphingosine reductase. CerS: dihydroceramide synthase. DES: dihydroceramide desaturase. CDase: ceramidase. SM: sphingomyelin. SMS: sphingomyelin synthase. SMase: sphinglomyelinase. SphK1,2: sphingosine kinase. SPPase: sphingosine-1-phosphate phosphatase. S1P: sphingosine-1-phosphate. SPL: sphingosine-1-phosphate lyase. Spns2: spinster 2. S1PR1: sphingosine-1-phosphate receptor 1. The figure has been created with BioRender.com.

To our knowledge, this is the first study describing how PE affects sphingolipid metabolism at the feto-placental vasculature. Our study reveals a distinct alteration of the sphingolipid profile during preeclampsia, which includes (Figure 3): increased de novo biosynthesis, accumulation of dhSph, and augmented production of SM in chorionic arteries. In PE endothelial cells, the increased expression of Nogo-B, SPPase, and S1P lyase, together with the decreased S1PR1 expression and concomitant S1PR2 upregulation, indicate the onset of an endothelial dysfunction, typical of this disorder. In the present study, all the experiments were carried out exclusively on human tissues, which is the strength of our work. One limitation of our study is the small cohort. However, we believe that our study paves the way for future research on the role of sphingolipids in the pathogenesis of preeclampsia.

## 4. Materials and Methods

### 4.1. Study Population

In this study, preeclampsia was defined according to the guidelines of the American College of Obstetricians and Gynecologists (ACOG, 2019). Clinical characteristics for the PE study are summarized in the Supplementary Table S1. All subjects gave written informed consent. All experiments were performed in accordance with the protocols approved by the ethical committee of the Medical University of Graz (Vote no: 29-319 ex 16/17).

### 4.2. Isolation of Arterial Chorionic Vessels and Primary Human Placental Arterial Endothelial Cells (fPAEC)

Placentae from cesarean section and vaginal delivery were used within 20 min after delivery (*n* = 10/Control group, *n* = 8/PE). The amnion was removed and the arterial chorionic vessels with a length of ~3 cm were resected and washed in Hank’s balanced salt solution (HBSS, Gibco, Thermo Fisher Scientific, Carlsbad, CA, USA). Next, isolated arteries were snap frozen or fixed in PFA for further processing. fPAECs were isolated from arterial chorionic blood vessels, as firstly described by Lang et al. [66].

### 4.3. Sphingolipid Analysis by LC-MS/MS

Chorionic placental arteries homogenates from normotensive and preeclamptic donors were used for quantification of sphingolipids by LC-MS/MS. The levels of ceramide (Cer) species, sphingosine (Sph), and S1P were analyzed by the Lipidomics Analytical Core at the Medical University of South Carolina, as previously described [67]. Lipid extraction was performed according to Bligh and Dyer [68]. For quantitative analysis of sphingolipid, eight-point calibration curves were generated for each target analyte. Synthetic as well as internal standards were spiked into an artificial matrix, and then subjected to an identical extraction procedure as the biological samples. These extracted standards were subsequently analyzed by the LC-MS/MS system operating in positive multiple reaction-monitoring (MRM) mode employing a gradient elution. Results were then calculated by plotting the sample area ratios against their corresponding standard. The MS analysis represents the mass level of particular sphingolipid (in pmols) per total sample used for lipid extract preparation. For the final data presentation, MS results were normalized to total protein (mg).

### 4.4. SPT Activity Assay

SPT activity in placenta arteries was measured as previously described [69]. Briefly, placenta arteries were homogenized in SPT reaction buffer (0.1 M HEPES (pH 8.3 at 25 °C), 5 mM DTT, 2.5 mM EDTA (pH 7.4), 50 μM pyridoxal 5′-phosphate (PLP; Sigma)). The assay was conducted in a volume of 0.1 mL composed by 200 μg of protein lysates, 0.45 μM [3H] serine (PerkinElmer), 0.2 mM palmitoyl-CoA (Sigma). After 15 min at 37 °C, the reaction was stopped with NH4OH and the product 3-ketosphinganine converted into sphinganine with NaBH4 (5 mg/mL). Radiolabeled lipids were extracted by using a modified Bligh and Dyer’s method [68], dissolved in CHCl3, and analyzed by thin-layer chromatography.

### 4.5. Quantitative Real-Time PCR (qPCR)

fPAECs were washed twice in pre-warmed HBSS and harvested in 350 µL RLT Lysis buffer (Quiagen, Hilden, Germany) supplemented with 1% β-mercaptoethanol (Sigma Aldrich, St. Louis, MO, USA), whereas chorionic arteries were snap frozen in liquid nitrogen and homogenized in 400 μL TRIzol. Next, total RNA content from cells and tissue lysates was isolated using the RNeasy®® Mini Kit (Quiagen, Hilden, Germany). Reverse transcription was performed using 100 ng of RNA and Maxima Reverse Transcriptase (200 U/μL; Thermo Scientific, USA). For the real-time PCR analysis, SYBR green PCR Master Mix (Qiagen, Hilden, Germany) and iCycler Applied Biosystems 7700 were used. 18S and HPRT1 were used as housekeeping genes. Primers sequences used for the real time PCR are listed in Supplementary Table S2.

### 4.6. Immunostaining

Isolated arterial chorionic placental vessels were incubated in calcium-free Krebs for at least 30 min to allow vessel vasodilation. Subsequently, the arteries were fixed with 4% PFA and left overnight at 4 °C. PFA-fixed arteries were OCT-embedded. For immunofluorescence, frozen placental artery sections were stained for Nogo-B (1:200, R&D), S1PR1 (1:200, R&D), and CD31 (1:200, Invitrogen) overnight at 4 °C and were then stained with Cy5-labeled anti-goat antibody (#A21436, Invitrogen, 1:500) Alexa 488 anti-rabbit (#016-540-084, Jackson ImmunoResearch, 1:200) and Alexa 568 anti-mouse in PBS for 1 h. Nuclei were stained with DAPI. Confocal immunofluorescence images of the tissues were captured on an Olympus Fluoview confocal microscope and quantified with ImageJ.

### 4.7. Statistical Analysis

Data are expressed as mean ± SEM. Statistical analysis were run with unpaired Student’s *t*-test. Differences were considered statistically significant when *p* < 0.05. GraphPad Prism software (version 8.0, GraphPad Software, San Diego, CA, USA) was used for all statistical analysis.

## 5. Conclusions

In conclusion, to our knowledge, this is the first study to show the impact of PE on sphingolipid metabolism in the feto-placental vasculature. We demonstrated that in blood vessels of PE placentae, the sphingolipid biosynthesis is shifted towards sphingomyelin production rather than ceramide and, concomitantly, an impairment of the vasculoprotective S1P signaling was observed. Taken together, these results indicate a shift of the feto-placental vasculature towards a pathological state.

## Supplementary Materials

Supplementary materials can be found at https://www.mdpi.com/1422-0067/21/3/1019/s1.

## Abbreviations

S1P	Sphingosisne-1-phosphate
S1PR	Sphingosisne-1-phosphate receptor
Cer	Ceramide
SM	Sphingomyelin
UCA	Umbilical cord artery
fPAEC	Fetal placental arterial endothelial cells
SPT	Serine palmitoyltranferase
SPTLC1	Serine palmitoyltranferase long chain base subunit 1
SPTLC2	Serine palmitoyltranferase long chain base subunit 2
SPNS2	Spinster transporter 2
SPHK1	Sphingosine kinase 1
SPHK2	Sphingosine kinase 2
SGPP1	Sphingosisne-1-phosphate phosphatase 1
SGPL1	Sphingosisne-1-phosphate lyase 1
Nogo-B	Reticulon-4B
