# Supplementary material for: Sphingolipid Signature of Human Feto-Placental Vasculature in Preeclampsia
Source: Int J Mol Sci. 2020 Feb 4;21(3):1019. doi: 10.3390/ijms21031019 (PMC7037072; doi:10.3390/ijms21031019)
Supplement: Supplementary file 1 [file ijms-21-01019-s001.pdf]

Supplementary Material

Sphingolipid signature of human feto-placental vasculature in preeclampsia

Ilaria Del Gaudio [1, 2], Linda Sasset [2], Annarita Di Lorenzo [2,*] and Christian Wadsack [1,*]

[1] Department of Obstetrics and Gynecology, Medical University of Graz, 8036 Graz, Austria; ilaria.del-gaudio@medunigraz.at; christian.wadsack@medunigraz.at

[2] Department of Pathology and Laboratory Medicine, Cardiovascular Research Institute, Feil Family Brain and Mind Research Institute, Weill Cornell Medical College, Cornell University, 10065 New York, New York, USA; lis2047@med.cornell.edu; and2039@med.cornell.edu

* Correspondence: christian.wadsack@medunigraz.at; Tel.: +43-316-385-81074 (C.W.) and and2039@med.cornell.edu; +1-212-746-6476 (A.D.L.)

	PN	PE	*P* value
Age (years)	33±2.9	29.8±4.4	n.s.
Gestational age (weeks)	36.7±1.8	34.4±3.6	n.s
BMI (kg/m^2)	22.3±2.5	23.9±1.9	n.s
SBP (mmHg)	113.6±12.1	160.5±10.5	p = 0.0003***
DBP (mmHg)	76.3±7	103.4±10.3	p = 0.0004***
Proteinuria (mg/24h)	-	954.5±350.5	-
sFlt-1/PlGF ratio	-	323.1±211.9	-
Placental weight (g)	680±156.3	415.7±104.4	p = 0.0015**
Fetal PI (kg/m^3)	2.5±0.2	2.4±0.2	n.s

Supplementary Table 1. Subjects characteristics of collected placentae for chorionic arteries and fPAECs isolation. Abbreviations: BMI = body-mass index; SBP = systolic blood pressure; DBP = Diastolic blood pressure; sFlt= Soluble fms-like tyrosine kinase 1(pg/ml); PlGF= Placental growth factor (pg/ml); PI= Fetal ponderal index is a comparable of BMI in adult subjects but relates weight to the cubed body length. All data are presented as mean±SD.

Probe	Forward (5′-3′)	Reverse (5′-3′)
SPTLC1	AGTGGGTTCTGGTGGAGATG	CAAGAGGTTCTGGTTGCCAC
SPTLC2	CCCCTGACCAAGTTACCTGT	TCGCTTCACAGTCATCCAGT
SPHK1	CCTGGGCAGTGAGATGTTTG	CTGCAAACACACCTTTCCCA
SPHK2	GACAGAACGACAGAACCACG	AGGCATCTTCACAGCTTCCT
SGPP1	GATCTGGAACCTGGACCCTC	ATAGAAATGGGGATGGCGGT
SGPL1	CGAGATGACAAATGGGGCAG	GCGACCTATTGAAGTGCCTG
SPNS2	GAAGGCCCTGATTCGAAACC	CGCCCAGAAATCCCGTAAAG
S1PR1	CGGCATTACAACTACACGGG	CCCCAGACAAGAGCAGGTTA
S1PR2	TTTTCTTCCCTGGCCCCTAG	TGTGGATTTGGGCTCTGGAT
S1PR3	CTGCTGACATCATCCACTGC	GCATCCATTCTACGCACAGG
RTN4B (Nogo-B)	CCTGCTCTCTGTGACCATCA	GCGCCTGAGTTCCTTTATCG
18S	CTACCACATCCAAGGAAGCA	TTTTTCGTCACTACCTCCCCG
HPRT1	CATTATGCTGAGGATTTGGAAAGG	CTTGAGCACACAGAGGGCTACA

Supplementary Table 2. Primers sequences for real-time PCR
